# Oral Microbiome of Deep and Shallow Dental Pockets In Chronic Periodontitis

**DOI:** 10.1371/journal.pone.0065520

**Published:** 2013-06-06

**Authors:** Xiuchun Ge, Rafael Rodriguez, My Trinh, John Gunsolley, Ping Xu

**Affiliations:** 1 VCU Philips Institute of Oral and Craniofacial Molecular Biology, Virginia Commonwealth University, Richmond, Virginia, United States of America; 2 Department of Periodontics, School of Dentistry, Virginia Commonwealth University, Richmond, Virginia, United States of America; University of Florida, United States of America

## Abstract

We examined the subgingival bacterial biodiversity in untreated chronic periodontitis patients by sequencing 16S rRNA genes. The primary purpose of the study was to compare the oral microbiome in deep (diseased) and shallow (healthy) sites. A secondary purpose was to evaluate the influences of smoking, race and dental caries on this relationship. A total of 88 subjects from two clinics were recruited. Paired subgingival plaque samples were taken from each subject, one from a probing site depth >5 mm (deep site) and the other from a probing site depth ≤3mm (shallow site). A universal primer set was designed to amplify the V4–V6 region for oral microbial 16S rRNA sequences. Differences in genera and species attributable to deep and shallow sites were determined by statistical analysis using a two-part model and false discovery rate. Fifty-one of 170 genera and 200 of 746 species were found significantly different in abundances between shallow and deep sites. Besides previously identified periodontal disease-associated bacterial species, additional species were found markedly changed in diseased sites. Cluster analysis revealed that the microbiome difference between deep and shallow sites was influenced by patient-level effects such as clinic location, race and smoking. The differences between clinic locations may be influenced by racial distribution, in that all of the African Americans subjects were seen at the same clinic. Our results suggested that there were influences from the microbiome for caries and periodontal disease and these influences are independent.

## Introduction

In the oral ecosystem, many microbial species exist in dental biofilms on both hard and soft tissue oral surfaces. A wide variety of bacterial types have been reported with more than 700 species present in the oral community [1]. Strong evidence suggests that oral microbes comprise a complex community [2]–[6]. Additionally, the composition and complexity of the biofilm depends on host responses, oral diseases and physical location in the oral cavity [7]–[9]. For these reasons the study of the oral ecosystem must take into account these factors. The ecosystem varies due to oral pathological processes such as periodontal disease and caries increasing the complexity and composition of the biofilm [10]–[12]. For example, carious lesions contain many acid-tolerant organisms including streptococci, lactobacilli, *Actinomyces*, *Prevotella*e and *Candida* yeasts [13].

Polymicrobial biofilms related to oral infectious diseases are prevalent in humans. Up to 90% of the population worldwide is affected by periodontal diseases [14]. The primary etiology of periodontal disease is plaque biofilm affecting the periodontal tissues. Dental plaque is divided into supragingival and subgingival plaque. Supragingival plaque has been generally associated with dental decay and subgingival plaque (plaque within the periodontal pocket) has been associated with periodontal diseases [15]. However, bacterial types associated with periodontitis can be found in both supra and subgingival plaque samples, but in very different proportions [16], [17]. Control of the periodontal biofilm with professionally administered periodontal therapy can slow or stop periodontitis and tooth loss for many years [18].

As the next generation sequencing technology development, NIH has funded an initiative Roadmap research to study associations of microbiome and human health/disease [19], [20]. Microbiome can be profiled using pyrosequencing 16S rRNA genes to study changes of microbial taxa in human body sites [21]. Here we present a study of oral microbial communities in subjects with periodontal disease using this technology. Bacterial samples were collected and sequenced from areas of relative periodontal health (pockets of 3 mm or less) and areas of disease involvement (greater than 5 mm) in patients with chronic adult periodontitis. Bacteria were characterized and classified using pyrosequencing of 16S rRNA genes. Our findings shed new light on the relationship between the oral microbiome and periodontal disease.

## Results

### 1. Patient Distribution

To reduce patient-to-patient variation in testing for differences in the microbiome between deep and shallow pockets, we obtained paired samples from each subject. Samples were obtained one from deep (diseased) site and one from shallow (healthy) site from each subject. The paired design reduces variation between samples from diseased and non-diseased sites, since each patient contributes a sample from both an area of periodontal health and an area of disease.

We recruited 92 subjects with different levels of chronic periodontitis, 88 of whom were successfully characterized at both sites. These subjects were recruited at two different clinic locations: Virginia Commonwealth University (VCU) in Richmond, VA and a Mission of Mercy project held in Wise, VA. The demographic and clinical parameters were summarized in Table 1. The average age of the participants was 47.8 years, ranging from 25 to 71 years old. Fifty-six of the subjects were current smokers with a mean pack-year history of 30.8. The probing depth and clinical attachment level for the deep sites sampled were 6.1±1.5 mm and 6.0±2.1mm, respectively. The corresponding clinical values for the shallow sites were 2.6±0.6 mm and 2.8±1.7 mm, respectively. Bleeding on probing was noted for 84.1% of the deep and 29.5% of the shallow sites sampled. The distribution of subjects was different at the two locations. At VCU, the sample was equally distributed between African Americans and Caucasians (Table S1), however, the subjects at the Wise location were entirely Caucasians. Another difference was that the subjects from Wise smoked at a much higher level than those from VCU.

**Table 1:** Summary of patients and category.

Variables	Mean (SD)
Age	47.8 (9.7)
Race (African American/Caucasian)	11/77
Gender (female/male)	36/52
Smokers (yes/no)	56/32
Packages per year for smokers	33.8*
Periodontitis (GMCP/GMLSCP/GSCP)	19/47/22
Caries risk (low/moderate/high)	11/58/19
Probing depth (mm; shallow sites)	2.6 (0.6)
Probing depth (mm; deep sites)	6.1 (1.5)
Clinical attachment level (mm; shallow sites)	2.8 (1.7)
Clinical attachment level (mm; deep sites)	6.0 (2.1)
Bleeding on probing (%; shallow sites)	29.5
Bleeding on probing (%; deep sites)	84.1

* , except for 3 patients chewing tobacco.

### 2. Sequence Profile Analysis of the Oral Microbiome

The V3 and V6 regions of 16S rRNA genes have been shown to possess a high degree of variability [22]. Because the length limit of current 454 DNA sequencing is ∼500 bases, we decided to use the V4–V6 regions (∼566 bp) for pyrosequencing. We designed barcoded primers to bind to conserved flanking regions (see Table S2). To allow amplification of sequences from as many oral bacterial species as possible, we downloaded the collection of oral bacterial 16S rRNA genes from the Human Oral Microbiome Database (HOMD v11.0) [2]. All oral bacterial 16S rRNA sequences were aligned and consensus sequences were examined within the selected conserved regions. To recover more oral bacterial species, degenerate sequences were introduced into the primers. Primers OM_515F and OM_1061R were designed as “universal” primers for the oral microbiome (Table S3). Coincidentally, they were identical to some previously published 16S primers (Table S3). We compared the 16S primers by searching the oral microbial 16S rRNA gene database. OM_1061R primer covered at least 30 more oral microbial strains than other primers at the same location (comparing with 1061R [23], 1052–1071 [24] or 1053–1068 [25] in Table S3). The missing microbial genera included *Aggregatibacter, Enterobacter, Escherichia, Fusobacteria, Haemophilus, Klebsiella, Kluyvera, Leptotrichia, Proteus, Sneathia, Terrahaemophilus,* and *Yersinia* (Table S4). OM_515F covered the same number of species as other primers at the same location (comparing with 515–532 [24] or 515–534 [25] primer in Table S3) although it contained no degenerated bases. GS FLX Titanium ‘Primer A’ sequence was added to the 5′ end of the OM_1061R primer for 454 sequencing (Table S2).

To perform sequencing in a cost-effective manner, we introduced 6-base barcodes to the OM primers. This produces 4^6^ = 4096 different sequences, each of which could be used to differentially tag sequences derived from different samples, allowing for their simultaneous analysis in the same sequencing run. We considered the following parameters when designing the barcodes. (1) All homodinucleotides (AA, GG, CC, and TT) were disallowed, (2) GC percent was restricted to 50% to avoid Tm changes among barcodes. (3) At least two base differences between any two barcodes were required to account for sequencing errors (Table S2).

**Figure 1:**
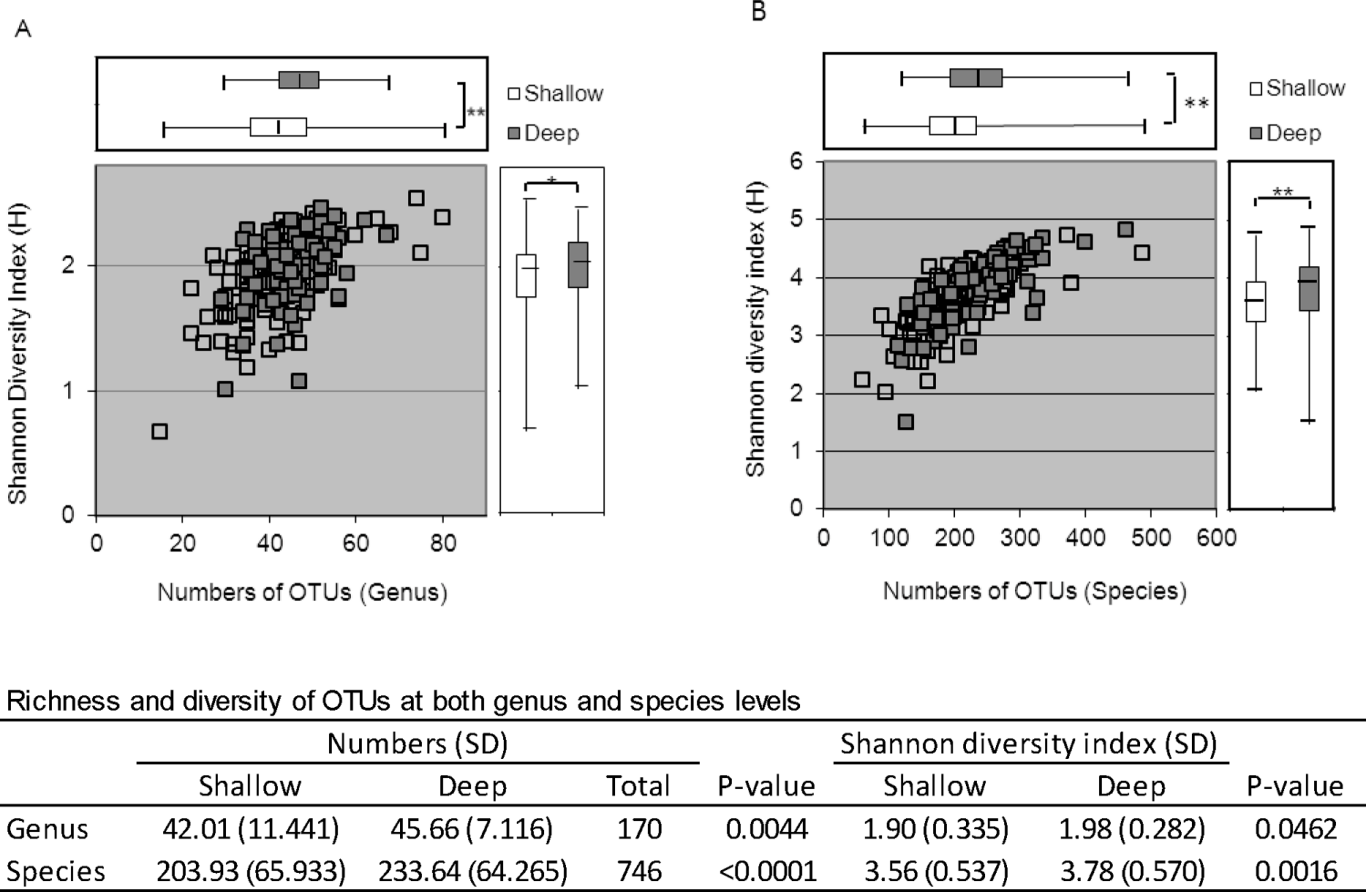
Shannon diversity index of genus- (A) and species-level (B) OTUs in the deep and shallow sites. *, p-value<0.05; **, p-value<0.01.

**Figure 2:**
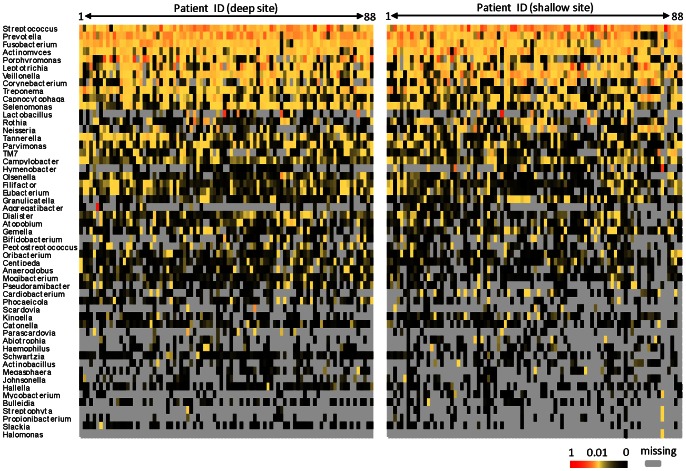
Genus-level OTUs with >1.0% of abundance in at least one patient in deep and/or shallow sites. Colors reflect the abundance of OTUs from high (red) to low (black); gray, OTU missing.

We collected a total of 2,299,090 sequences, 1,790,237 of which qualified sequences were considered (as defined as described in Materials and Methods) and were analyzed further. All raw sequences were deposited in the NCBI Sequence Read Archive (access number SRA061999). The average was about 10,000 sequences per sample. Sequence barcoding indexes were associated with patient samples for analysis. Sequences were grouped for genera by operational taxonomic units (OTUs) using Ribosomal Database Project (RDP) Classifier 2.2. Microbial species were classified using BLAST search against HOMD 16S rRNA database. A total of 170 and 746 independent microbial genera and species were identified within the samples analyzed. For every deep and shallow site sampled, the percentage of each individual genus and species from the overall microbial abundance was calculated. The richness and diversity of OTUs at both genus and species levels were analyzed by the Shannon diversity index [26] and paired t-test (Figure 1). The results indicated that the microbiome in deep sites had higher numbers of both genera and species and a greater range of diversity values than shallow sites, and this difference was statistically significant (P<0.05). Based on genus-level OTUs, the dominant genera (average abundance >1.0% of the total per subjects) included *Streptococcus, Prevotella, Fusobacterium, Actinomyces, Porphyromonas, Leptotrichia, Veillonella, Corynebacterium, Treponema, Capnocytophaga, Selenomonas, Lactobacillus, Rothia, Tannerella,* and *Parvimonas* in both deep and shallow sites (Figure 2). There were 35 other genera present at an abundance >1.0% of the population in at least one patient each (Figure 2).

### 3. Microbiome Associated with Chronic Periodontitis

**Figure 3:**
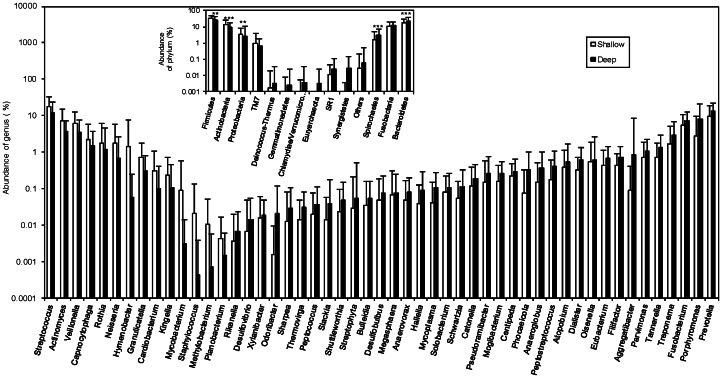
Genus-level OTUs significantly different between shallow and deep pockets. Of 170 genera, 51 showed a statistically significant difference in abundance (%) between shallow and deep sites. Data were statistically analyzed by the two-part model and false discovery rate (FDR). The FDR q-value of 51 genera was <0.05 which represents significant difference between shallow and deep samples. Inserted figure, phylum-level OTUs significantly different between shallow and deep sites; **, p-value <0.01; ***, p-value <0.001.

The difference in abundance of OTUs between the shallow and deep sites was statistically analyzed at genus and species levels using a two-part model and a false discovery rate (FDR). Fifty-one out of 170 genus-level OTUs were shown to be significantly different in abundance between shallow and deep sites (Figure 3). In the deep sites, 14 genus-level OTUs, including *Streptococcus*, *Actinomyces* and *Veillonella*, were decreased in abundance, whereas 37 genus-level OTUs were present in increased abundance compared to shallow sites. The OTUs with increased abundance in deep sites included not only previously identified pathogens of periodontal diseases such as *Prevotella*, *Porphyromonas*, *Treponema* and *Fusobacterium* but also many other previously unreported bacterial genera such as *Phocaeicola*-. Dominant bacteria, such as *Streptococcus* and *Prevotella* (average abundance >10%), were present in high levels in both deep and shallow sites. At the species level, there were 200 OTUs statistically different between shallow and deep sites (Table S5). Among these OTUs, 148 were shown to be significantly more abundant in deep sites compared to shallow sites. *Porphyromonas gingivalis*, *Porphyromonas endodontalis*, *Fusobacterium nucleatum*, *Prevotella nigrescens*, *Treponema denticola*, *Treponema medium*, *Tannerella forsythia* and *Parvimonas micra* were the representative species of genera *Porphyromonas*, *Fusobacterium*, *Prevotella*, *Treponema*, *Tannerella* and *Parvimonas* exhibiting increases in deep sites. We also found significantly greater abundance of 52 other species-level OTUs in shallow sites (Table S5). The OTUs with higher abundance in shallow sites included *Streptococcus oralis*, *Streptococcus sanguinis*, *Streptococcus gordonii, Rothia dentiocariosa*, *Veillonella dispar*, *Actinomyces naeslundii* and *Actinomyces* sp.

**Figure 4:**
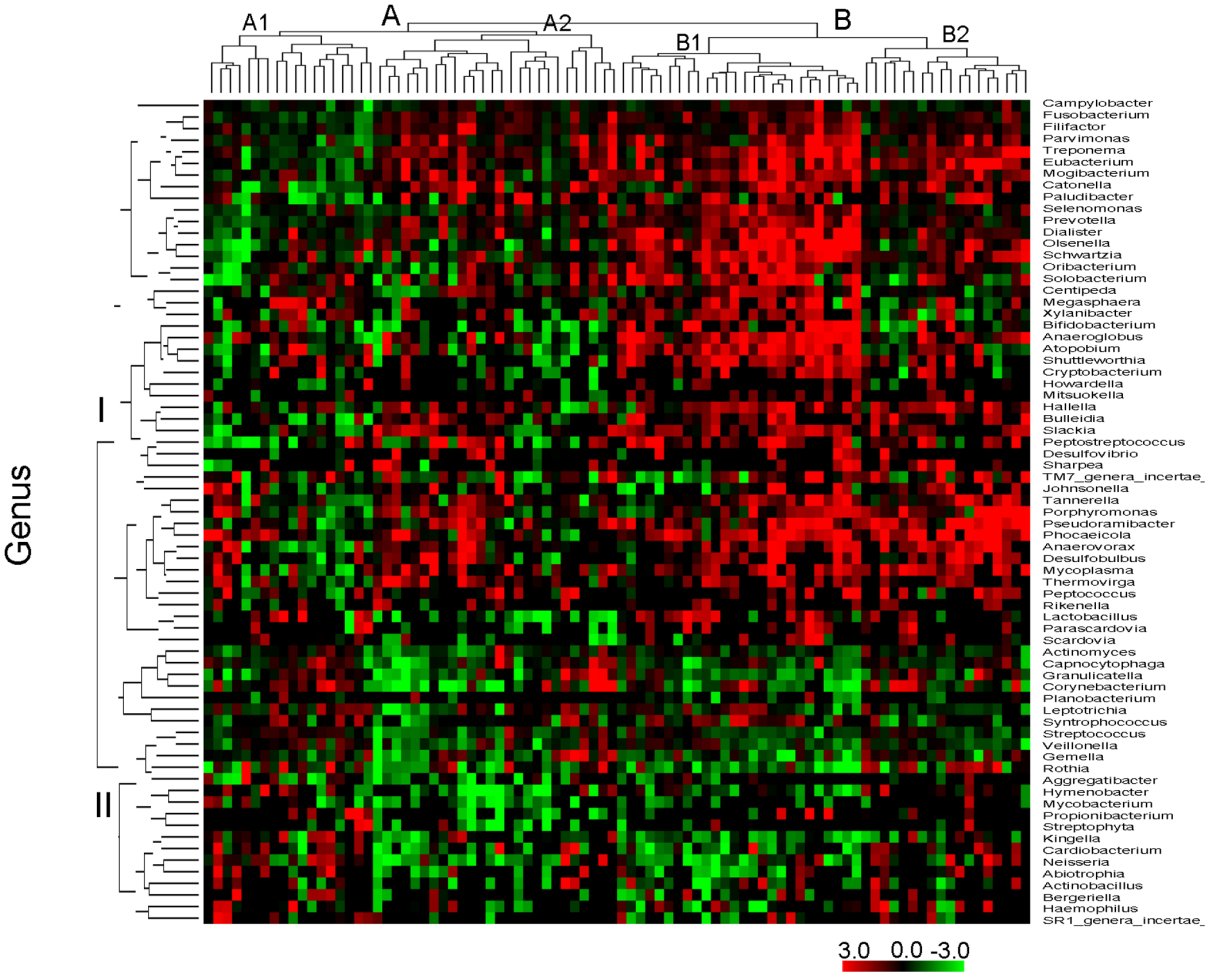
Clustering analysis of log_2_ ratio of deep abundance to the shallow at genus level using Hierarchical Trees (HCL). The data were obtained using log_2_ ratio (deep abundance/shallow abundance). Both deep and shallow abundance had 0.00001 added due to the many zeros in the samples. The genera present in fewer than 10% of samples were excluded. The genera were clustered into two groups (I and II) and 88 patients were grouped into two types (A and B), each of which contains 2 subtypes (A1 and A2, B1 and B2).

To cluster the microbiome in the 88 subjects, the log_2_ ratios of abundance per OTU in deep sites to that of shallow sites in the same patient were obtained. After analysis by Hierarchical trees clustering (HCL), the results showed that 71 genera, which were present in at least 10% of samples, could be clustered into two groups I and II (Figure 4). The majority of genus-level OTUs which were increased in deep sites (including *Prevotella*, *Porphyromonas*, *Treponema* and *Fusobacterium)* were clustered in group I, while genus-level OTUs which were decreased in deep sites (including *Streptococcus*, *Actinomyces* and *Veillonella*) were clustered in group II. At the species-level, a similar pattern was found, with two major clusters with most OTUs increased in deep sites in one cluster and decreased in deep sites in the other cluster (Figure S1). Based on the abundance log_2_ ratio of the deep to the shallow in each patient, the patients were also clustered by HCL. For the cluster of microorganisms at genus level, the 88 patients could be grouped into types A and B with 44 patients each (Figure 4). Each type could be further divided into 2 subtypes (A1 and A2, B1 and B2).

In order to determine if patient factors influenced the log_2_ ratio of differences between deep and shallow sites, the clusters were evaluated for consistency of these factors. The patient variables were, race, gender, smoking, location (the clinic site where the samples were collected) and classification by the extent of caries of the patient (three groups: none, moderate and high)(Table 2 and Table S6). The bivariate results in Table 2 from Chi-square analysis indicated that racial distribution, caries status and location differed significantly between the clusters. The proportions of type A in African American patients and VCU subjects were found to be significantly greater than those of type B (Table 2, Race and Region). Because all of the African Americans were seen at VCU, we tested the difference in proportions of two types between VCU and Wise after the African Americans samples were removed. The result showed there is no significant difference in proportions of two types between VCU and Wise (P = 0.515). This data suggested that the significant difference in proportions of types between the two locations might result from the significant difference in proportions of types between African Americans and Caucasians. After multivariate analysis between patient’s types and all factors using logistic regression, the factor of smoking besides location was shown to significantly affect the patient’s types (P = 0.0238; Table 2).

**Table 2:** Associations of patient types with other factors

		Type A			Type B			P-value1†	P-value2‡
		A1	A2	A1+A2	B1	B2	B1+B2		
Race	AA	4 (36.4)*	6 (54.5)	10 (90.9)	1 (9.1)	0 (0)	1 (9.1)	0.0037	0.0660
	Ca	14 (18.2)	20 (26)	34 (44.2)	25 (32.5)	18 (23.4)	43 (55.8)		
Location	VCU	9 (36)	10 (40)	19 (76)	5 (20)	1 (4)	6 (24)	0.0021	0.0495
	Wise	9 (14.3)	16 (25.4)	25 (39.7)	21 (33.3)	17 (27)	38 (60.3)		
Gender	F	7 (19.4)	12 (33.3)	19 (52.8)	11 (30.6)	6 (16.7)	17 (47.2)	0.6646	0.9591
	M	11 (21.2)	14 (26.9)	25 (48.1)	15 (28.8)	12 (23.1)	27 (51.9)		
Smoking	non-smoking	3 (9.4)	11 (34.4)	14 (43.8)	11 (34.4)	7 (21.9)	18 (56.3)	0.3754	0.0238
	Smoking	15 (26.8)	15 (26.8)	30 (53.6)	15 (26.8)	11 (19.6)	26 (46.4)		
Caries	none	4 (21.1)	4 (21.1)	8 (42.1)	3 (15.8)	8 (42.1)ξ	11 (57.9)	0.7285	0.9533
	moderate	12 (20.7)	18 (31)	30 (51.7)	19 (32.8)	9 (15.5)	28 (48.3)		
	high	2 (18.2)	4 (36.4)	6 (54.5)	4 (36.4)	1 (9.1)	5 (45.5)		

* The number of subjects in each category is indicated. Numbers in parentheses are the percentage of the each (sub)type out of the total.

† Chi-square test for association of types A and B with each factor separately.

‡ Multivariate analysis between types and all factors using logistic regression.

ξ P-value = 0.037, for comparison of type B2 to others in dental caries.

### 4. Microbiome Associated with Dental Caries

**Figure 5:**
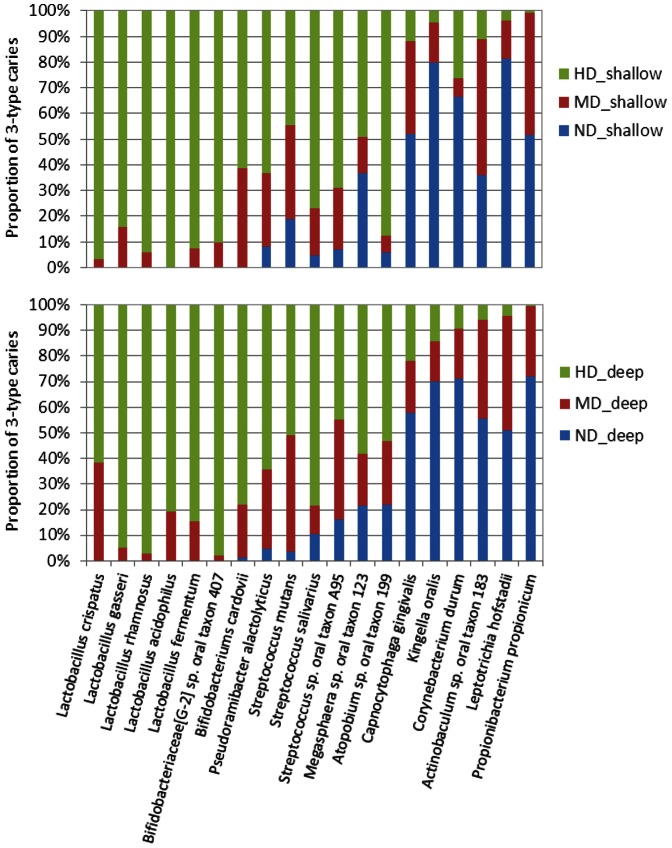
The species associated with dental caries. The abundances of species-level OTUs were statistically analyzed in 3 levels of dental caries in deep and shallow sites, respectively. The species with a significant difference (α = 0.05) in 3 levels of dental caries were shown. HD_shallow, high decay and shallow site; MD_shallow, moderate decay and shallow site; ND_shallow, non-decay and shallow site; HD_deep, high decay and deep site; MD_ deep, moderate decay and deep site; ND_ deep, non-decay in deep site.

The 88 patients with chronic periodontitis were also categorized according to dental caries status from non-decay to severe decay. We found that the genus-level proportions of subtype B2 in Table 2 decreased with increase in the severity of decay, compared with the other three subtypes. The caries status was found to be significantly different from that of other subtypes (P = 0.037). Based on three different levels of caries risk, we also statistically analyzed the difference in species-level OTUs of deep and shallow sites among these groups using Kruskal–Wallis test to uncover which bacterial species were potentially associated with dental caries. As shown in Table 3, there were 13 species-level OTUs whose abundances increased and 6 OTUs whose abundance decreased from no caries to high caries in both deep and shallow sites. There were 5 *Lactobacillus* spp. out of 13 OTUs that rose in moderate/high caries, 4 of which were not present in non-decayed subjects but appeared in moderate and high decayed subjects. Three streptococcal species including *Streptococcus mutans*, *Streptococcus salivarius* and *Streptococcus* sp. oral taxon A95 were the dominant OTUs that were increased in the moderate/high caries patients. For OTUs which abundances decreased, *Leptotrichia hofstadii* and *Kingella oralis* were the dominant OTUs in the non-decay patients. These results indicated that many bacterial species may be associated with dental caries in periodontitis patients, with *S. mutans* and *Lactobacillus* spp. being the most heavily associated with decay. It should be noted that of the 13 species that were higher in the subjects with moderate or high decay, none had levels different between deep and shallow pockets. The independence of the microbiome associated with periodontal disease and caries was also observed in Figure 5, which showed a similar proportional pattern of the caries-associated species in deep and shallow sites.

**Table 3:** Association of species-level microbiome with dental caries in deep and shallow sites.

Species	ND_shallow	MD_shallow	HD_shallow	P shallow	ND_deep	MD_deep	HD_deep	P deep	Change
Atopobium sp. oral taxon 199	1.95E-04	2.13E-04	2.87E-03	4.13E-02	2.02E-04	2.32E-04	4.92E-04	1.61E-02	Increase
Bifidobacteriaceae[G-2] sp. oraltaxon 407	0.00E+00	3.00E-06	2.80E-05	3.07E-02	0.00E+00	3.38E-06	1.58E-04	2.65E-02	Increase
Bifidobacterium cardovii	0.00E+00	2.76E-04	4.40E-04	2.89E-02	7.91E-06	1.27E-04	4.84E-04	1.64E-02	Increase
Lactobacillus acidophilus	0.00E+00	0.00E+00	4.75E-04	3.80E-03	0.00E+00	8.21E-05	3.48E-04	5.86E-03	Increase
Lactobacillus crispatus	0.00E+00	5.05E-05	1.55E-03	4.92E-03	0.00E+00	1.38E-03	2.24E-03	1.29E-02	Increase
Lactobacillus fermentum	0.00E+00	5.65E-04	6.96E-03	4.04E-02	1.65E-05	8.65E-04	4.84E-03	1.02E-03	Increase
Lactobacillus gasseri	0.00E+00	2.67E-04	1.45E-03	4.06E-02	0.00E+00	1.96E-04	3.52E-03	1.92E-02	Increase
Lactobacillus rhamnosus	0.00E+00	6.96E-05	1.08E-03	3.07E-02	0.00E+00	2.95E-05	1.04E-03	4.06E-02	Increase
Megasphaera sp. oral taxon 123	1.34E-03	5.09E-04	1.80E-03	2.52E-02	7.50E-04	7.12E-04	2.04E-03	3.31E-02	Increase
Pseudoramibacter alactolyticus	3.47E-04	1.23E-03	2.74E-03	3.88E-02	3.44E-04	2.36E-03	4.85E-03	1.17E-02	Increase
Streptococcus mutans	8.19E-03	1.58E-02	1.93E-02	2.97E-02	7.53E-04	9.25E-03	1.04E-02	1.32E-02	Increase
Streptococcus salivarius	6.19E-04	2.45E-03	1.03E-02	2.69E-02	1.14E-03	1.22E-03	8.68E-03	4.78E-03	Increase
Streptococcus sp. oral taxon A95	8.46E-04	2.99E-03	8.55E-03	4.77E-03	2.00E-03	4.90E-03	5.59E-03	2.78E-02	Increase
Actinobaculum sp. oral taxon 183	3.21E-03	4.77E-03	9.77E-04	4.66E-02	3.28E-03	2.27E-03	3.40E-04	8.92E-03	Decrease
Capnocytophaga gingivalis	4.43E-03	3.09E-03	1.02E-03	4.79E-03	6.28E-03	2.22E-03	2.38E-03	4.15E-02	Decrease
Corynebacterium durum	6.62E-03	6.93E-04	2.63E-03	5.90E-03	9.15E-04	2.55E-04	1.18E-04	2.39E-02	Decrease
Kingella oralis	1.14E-02	2.24E-03	6.77E-04	2.96E-03	2.77E-03	6.20E-04	5.60E-04	2.42E-02	Decrease
Leptotrichia hofstadii	2.22E-02	4.04E-03	1.03E-03	8.07E-03	4.13E-03	3.63E-03	3.67E-04	4.32E-02	Decrease
Propionibacterium propionicum	1.38E-03	1.27E-03	2.40E-05	1.31E-03	1.37E-03	5.18E-04	1.04E-05	2.56E-03	Decrease

ND, non-decay; MD, moderate decay; HD, high decay.

Change from ND to HD.

P, p value.

## Discussion

The oral microbiome varies dynamically over time. It may change from human daily activities such as brushing teeth, drinking different juices, smoking, eating etc. Due to the variation, it would be difficult to associate causative pathogens with periodontal diseases from a small group of subjects. We have examined microbial occurrence simultaneously by comparing deep and shallow sites of the same patients. In this way, the statistical power for our microbiome analysis was significantly increased. This method has also proved useful in previous studies [27]. While we clustered the OTUs abundance in deep and/or shallow sites of patients to understand the possible patterns of the microbiome and patients and their relationship, the results showed that the patterns of the microbiome and/or patients are ambiguous (data not shown). However, when we used the log ratio of abundance per OTU in deep site to that of shallow site in the same patient for clustering, the groups of microbiome and the types of patients were clearly revealed (Figure 4 and Figure S1). This analysis may benefit from the increased statistical power for microbiome analysis obtained by comparing paired deep and shallow sites in the same patient.

Our results revealed dominant bacterial phyla, such as *Firmicutes*, *Actinobacteria*, *Proteobacteria*, *Fusobacteria*, *Bacteroidetes* and *Spirochaetes* in healthy and diseased sites (Figure 3). This bacterial community is largely consistent with previous studies [2], [6]. In our study, *Firmicutes, Actinobacteria* and *Proteobacteria* exhibited significant reductions in diseased sites whereas *Bacteroidetes* and *Spirochaetes* increased markedly. Liu et al. (2012) identified 128 genera in 2 chronic periodontitis patients and 3 healthy individuals using 16S rDNA pyrosequencing. We identified 170 genera in diseased and healthy sites in periodontitis patients. The increased number of genera identified may result from the greater number of patient samples sequenced. Liu et al. (2012) did not detect *Fusobacterium* and *Porphyromonas*, implicated previously in periodontitis, as being significantly more abundant in their periodontal disease samples compared to healthy individuals. In our study, these two genera had significantly greater abundance in diseased sites than in healthy sites (Figure 3). For species-level OTUs, we compared microbial abundance changes between shallow and deep sites and identified statistically significant species difference (Table S5). We found that some of bacterial species, frequently associated with periodontal diseases, were significantly increased in diseased sites compared to the healthy sites (Figure S1), for example, *Porphyromonas gingivalis, Fusobacterium nucleatum, Prevotella intermedia, Prevotella nigrescens, Aggregatibacter actinomycetemcomitans, Tannerella forsythia,* and *Treponema denticola* [10]–[12]. But some of bacterial species such as *Streptococcus sanguinis, Streptococcus oralis, Streptococcus gordonii, Actinomyces* sp., *Rothia dentiocariosa* and *Veillonella dispar* were decreased in diseased sites. Many new species were listed in the table that may be used for association of periodontal diseases (Table S5). Thus, our study suggests that the pyrosequencing technology may identify additional bacterial types associated with periodontal disease.

Our results indicated that similar proportional pattern of caries-associated bacteria were present (Figure 5) in both deep and shallow sites, but varied according to the caries levels of the subjects. Thus, the occurrence of bacterial types associated with caries appears to be independent of the periodontal condition of the site. Using 16S rRNA gene sequencing technology, a recent study examined the bacterial community in dental caries in very young children [28]. They found that streptococci other than *S. mutans* and *Veillonella* may be also associated with caries in young children. In our study, *S. mutans*, *S. salivarius* and *Streptococcus* sp. oral taxon A95 were identified at higher levels in moderate/high caries patients compared to those of no-decay individuals (Figure 5). In addition, 5 *Lactobacillus* spp. were found significantly increased in moderate/high caries patients. *Bifidobacterium cardovii*, *Bifidobacteriaceae*[G-2] sp. oral taxon 407, and *Pseudoramibacter alactolyticus* were also identified at higher levels in moderate/high caries patients. These results support that bacteria besides *S. mutans* may be involved in dental caries.

Our results also suggested that it is difficult to predict periodontal disease on the basis of the mere presence of any bacterial species. For example, we found many bacterial species that were present in over 90% of samples including *Streptococcus, Prevotella, Actinomyces, Fusobacterium, Veillonella, Treponema, Leptotrichia, Tannerella* and *Porphyromonas*. Our data suggest that periodontal disease may be associated with a consortium of microbial species (or cell populations) rather than one or few specific bacterial species.

## Materials and Methods

### 1. Clinical Procedures

Subject recruitment was conducted with the approval of the VCU Institutional Review Board (IRB number HM12201). Written consent was obtained from all participants. The data were analyzed anonymously. The study was a cross-sectional study where two samples of plaque were obtained from each of 88 patients. The subjects were enrolled at either the VCU School of Dentistry or the MOM project at Wise, Virginia. The patients in the study satisfied the following inclusion/exclusion criteria: The inclusion criteria were: 1) good general health 2) ages of 25 to 71 (male or female) 3) five sites with probing depths (PD) of >5 mm in 5 non-adjacent interproximal spaces. 4) At least one interproximal area with PD ≤3 mm, so one site could be sampled as a periodontally healthy site. Individuals were excluded who were pregnant, had recent periodontal therapy (within three months), received antibiotic therapy in the past three months, who had systemic conditions which could influence the course of periodontal disease, and patients with aggressive forms of periodontal disease. The latter group was defined as subjects less than 35 years of age with eight or more teeth with severe periodontal disease. We also broadly classified the amount of dental caries in these individuals. Based on the severity of dental caries, 88 patients were characterized into 3 levels of dental caries: (1) non-decay, with no clinical detectable caries, (2) moderate decay (1–6 areas), with small areas of detectable decay and (3) high decay, with decay at most or all of the teeth consistent with rampant decay. It should be noted that this was a whole-mouth assessment and data for individual teeth were not obtained. The numbers of years that subjects had smoked and the number of packs were also characterized, so pack-years could be calculated. Current smokers were characterized as subjects who smoked at least one pack per day.

### 2. Sample Collection, PCR Amplification and DNA Sequencing

Plaque samples were obtained from deep (periodontal disease) and shallow (healthy) sites within each patient during the baseline appointment. Sterile gauze was used to isolate and dry the site to be sampled. Any supragingival plaque at the sampling site was removed with a cotton applicator. A sterile dental curette (e.g., Gracey 13/14, 15/16, 11/12, 7/8, 1/2) was inserted to the depth of the gingival sulcus, and a subgingival plaque sample was retrieved. Each sample was placed in a sterile, labeled tube containing 50 µL TE buffer [10 mM Tris-HCL, 1 mM ethylenediaminetetraacetic acid (EDTA), pH 7.6]. Genomic DNA from 176 samples of 88 subjects was isolated using PowerLyzer PowerSoil DNA isolation kit (Mo Bio) according to the manufacturer’s instructions.

Universal PCR primers were designed to amplify the V4–V6 variable rRNA regions (∼566 bp) [22]. To design the universal PCR primers for oral microbiome, we collected 755 oral bacterial 16S rRNAs in HOMD database [2] and compared their sequences to identify consensus sequences flanking V4–V6 regions. Degenerate sequences were further used in designing PCR primers to assure all oral strains were amplified. The final primers were OM_515F: 5′-GTGCCAGCAGCCGCGGTAA-3′ and primer OM_1061R: 5′-TCACRRCACGAGCTGWCGAC-3′. The pair of universal primers were synthesized to fit GS FLX Titanium ‘Primer A’ sequence including forward primer (5′ – CCATCTCATCCCTGCGTGTCTCCGACTCAG-3′) and reverse primer (5′-CCTATCCCCTGTGTGCCTTGGCAGTCTCAG-3′). Barcode sequence tags of 6 bases were attached to the universal primer set for identification of different samples in pools of sequencing samples (Table S2).

The V4–V6 regions of 16S rRNA genes were PCR amplified using high fidelity Taq DNA polymerase (Invitrogen, Carlsbad, CA). The PCR amplification was performed at 94°C for 1 min, 30 cycles of 94°C for 30 sec, 54°C for 30 sec and 68°C for 1 min, and 72°C for 5 min. The amplicons were purified using PureLink 96 PCR purification kit (Invitrogen) and subjected to DNA sequencing in Roche Genome Sequencer FLX System as described by the manufacturer.

### 3. Sequence Data Classification

Sequence clarification was carried out using the packages of Roche Genome Sequencer FLX System. Raw DNA reads were collected and sequences were filtered by quality score 20. To remove contaminant or chimeric sequences, sequences shorter than 200 bp or longer than 650 bp after filtering were not included as qualified sequences. For genus-level microbiome classification, the sequences were searched using RDP Classifier against the 16S rRNA database. For species-level classification, all 16S rRNA sequences of oral microbial strains were downloaded to a local computer from the HOMD database. A 16S rRNA database was created for oral microbial strain classification. The BLAST search program was used to identify matching 16S rRNAs. Microbial strains were assigned based on the best matched sequences with a cut-off of 97% sequence identity. The strains were finally classified into species.

### 4. Data Statistical Analysis

To examine the diversity of OTUs at both genus and species levels, the genus-and species-level OTUs in each patient sample were analyzed by Shannon diversity index (S) [26], calculated as: ***S = −∑ p_i_ log (p_i_)*** where *p_i_* is the proportion of the entire abundance made up at the *i*th OTU in the sample. The number of OTUs and the Shannon diversity index in shallow and deep sites of patients were statistically analyzed by paired t-test.

To compare the microbiome between shallow and deep sites, we mainly applied two-part statistics with paired data [29] for OTU abundances containing many zeros. For each microbe (phylum, genus and species), the zero-containing abundances between shallow and deep sites were firstly analyzed by McNemar’s test. If this test showed difference in the proportions of zeros values, the two-part model was powerful and used in those data. If the abundances did not differ in the proportions of zero values, Wilcoxon pairs signed-ranks test would be more powerful and was applied instead of the two-part model. For the microbes without zero abundance in all samples, Wilcoxon pairs signed-ranks test was used directly. After two-part or Wilcoxon pairs signed-ranks test analysis, p-values were analyzed by FDR using R package q-value. The q-value of FDR <0.05 represents significant difference in microbe abundances between the shallow and deep sites.

To examine differences in the microbe abundances among the three categories of dental caries, the data on microbiome in the deep and shallow sites were analyzed by the Kruskal–Wallis test.

### 5. Microbiome and Patient Clustering

The microbiome and patients were clustered using HCL based on comparison of the microbiome abundances between the shallow and deep sites. We selected the microbes present in at least 10% samples at both genus and species levels for HCL. For each microbe per patient, the data were transformed into log_2_ ratio of the deep abundance to the shallow. Due to many zeros in the samples affecting division and log transformation, each deep and shallow abundance had 0.00001 added, which became the minimum possible abundance. The log_2_ ratios of microbes were run by HCL with correlation of similarity metric and complete linkage on microbiome and patient clusters. After HCL clustering, microbiome groups and patient types or subtypes were obtained. To examine the relationship between patient’s types/subtype and various possible factors including region, race, gender, smoking and dental caries status, the Chi-square test for bivariate analysis and logistic regression for multivariate analysis was applied using SAS software version 9.3. If the sample size was not large enough, Fisher’s exact test was applied.

## Supporting Information

Figure S1 **Clustering analysis for log_2_ ratio of deep abundance to the shallow at the bacterial species level using Hierarchical Trees.** The data were obtained using log_2_ ratio (deep abundance/shallow abundance). Each deep and shallow abundance had 0.00001 added due to the many zeros in the samples. The species was excluded when it was present in fewer than 10% of samples.
(TIFF)

Table S1 **Demographic Distribution of the subjects.** The subject numbers, means and standard deviation (Std Dev) of age and smoke-pack years for location, gender and race were summarized. VCU, Virginia Commonwealth University; Wise, Wise, Virginia MOM project; F, female; M, male; AA, African American; CA, Caucasian.
(XLSX)

Table S2 **Primer sequences and barcodes for 16S rRNA pyrosequencing.** The primer 16S_F515 contains the reverse primer sequence for the GS FLX Titanium ‘Primer A’ and 16S rRNAs forward primer sequence (OM_515F). Each reverse primer is composed of the forward primer sequence for the GS FLX Titanium ‘Primer A’, a barcode sequence and 16S rRNAs reverse primer sequence (OM_1061R).
(XLSX)

Table S3 **Comparison of universal primers for oral microbial database.** The primer sequences from the references and this study were run BLASTN search in HOMD database. The matched species hints were shown for each primer.
(XLSX)

Table S4 **Microbial species recovered by OM_1061R but not 1061R.** The species and genera for primers OM_1061R and 1061R obtained from the BLSATN search in HOMD database were compared. The unique ones for OM_1061R were listed.
(XLSX)

Table S5 **Comparison of species-level microbiome between shallow and deep pockets.** The species-level OTUs between shallow and deep pockets were statistically analyzed by using a two-part model and false discovery rate (FDR). The p-values for species between deep and shallow sites yielded from the two-part model were performed on multiple comparisons by FDR. The q-value of FDR <0.05 represented significant difference in microbial abundances between the shallow and deep sites. The species with significant difference between the shallow and deep sites were shown. SD_shallow, standard deviation of shallow; SD_Deep, standard deviation of Deep; Change, difference between Deep and Shallow.
(XLSX)

Table S6 **Distribution of caries.** The subject numbers of three-type caries for location, gender and race were summarized. VCU, Virginia Commonwealth University; Wise, Wise, Virginia MOM project; F, female; M, male; AA, African American; CA, Caucasian.
(XLSX)
