# Supplementary figures and images for: Oral Microbiome of Deep and Shallow Dental Pockets In Chronic Periodontitis
Source: PLoS One. 2013 Jun 6;8(6):e65520. doi: 10.1371/journal.pone.0065520 (PMC3675156; doi:10.1371/journal.pone.0065520)

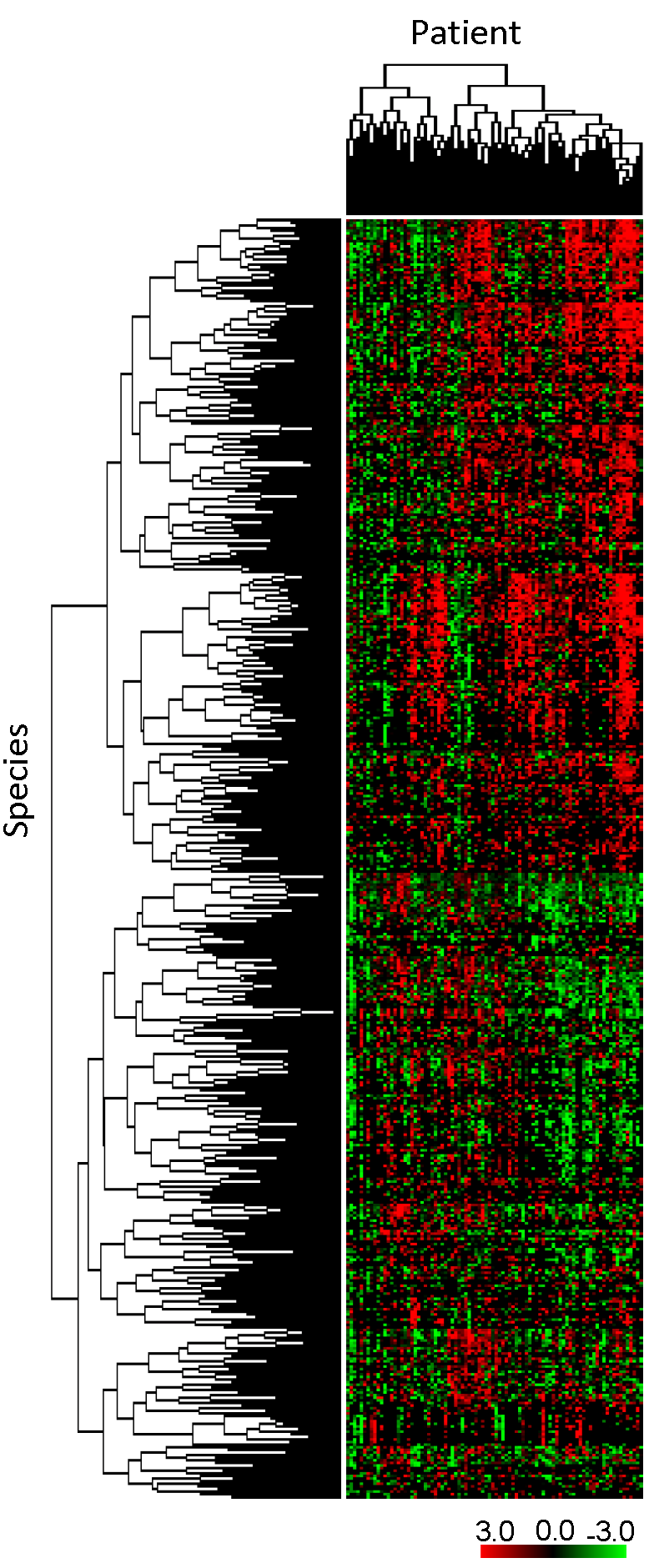

Supplement: Figure S1 — Clustering analysis for log2 ratio of deep abundance to the shallow at the bacterial species level using Hierarchical Trees. The data were obtained using log2 ratio (deep abundance/shallow abundance). Each deep and shallow abundance had 0.00001 added due to the many zeros in the samples. The species was excluded when it was present in fewer than 10% of samples. (TIFF) [file pone.0065520.s001.tif]
